# Expanding the genetic and clinical spectrum of SLC25A42‐associated disorders and testing of pantothenic acid to improve CoA level in vitro

**DOI:** 10.1002/jmd2.12441

**Published:** 2024-08-21

**Authors:** Katharina Heckmann, Arcangela Iuso, Janine Reunert, Marianne Grüneberg, Anja Seelhöfer, Stephan Rust, Giuseppe Fiermonte, Eleonora Paradies, Carmela Piazzolla, Manoj Mannil, Thorsten Marquardt

**Affiliations:** ^1^ Department of General Pediatrics University Hospital Münster Münster Germany; ^2^ Institute of Neurogenomics, Helmholtz Zentrum München Neuherberg Germany; ^3^ Institute of Human Genetics Technical University of Munich Munich Germany; ^4^ Department of Biosciences, Biotechnologies and Biopharmaceutics University of Bari Bari Italy; ^5^ Clinic of Radiology University Hospital Münster Münster Germany

**Keywords:** cellular CoA, mitochondrial coenzyme transporter, mitochondrial respiration, pantothenic acid, SLC25A42

## Abstract

*SLC25A42* encodes the mitochondrial coenzyme A (CoA) transporter localized at the inner mitochondrial membrane. SLC25A42 deficiency leads to a congenital disease with a heterogeneous clinical presentation, including myopathy, developmental delay, lactic acidosis, and encephalopathy. Twenty‐one patients have been described so far. In the current study, we report on the identification of new biallelic variants in *SLC25A42* in three siblings. Patients presented with symmetrical T2 hyperintensity of the putamen with minor volume depression at the brain MRI, elevated lactate, reduced oxygen consumption rates in muscle and fibroblasts, and reduced CoA levels in fibroblasts. Administration of pantothenic acid led to clinical stabilization and increased CoA levels in fibroblasts, thus confirming a role for SLC25A42 in energy metabolism and CoA homeostasis.

**Synopsis**

New mutations in *SLC25A42* lead to reduced CoA levels and treatment with pantothenic acid in fibroblasts leads to CoA normalization.

## 1 INTRODUCTION

The solute carrier family 25 member 42 gene (*SLC25A42*, OMIM * 610823) is located on chromosome 19 and is composed of seven exons encoding a 418 amino acids protein consisting of six transmembrane alpha‐helices, similar to other proteins of the solute carrier family 25 (SLC25).^1^ In rats, members of the SLC25 family are widely expressed in tissues, especially in the adipose tissue, hypothalamus, pons, and liver.^1^ Compared to the other SLC25 proteins, SLC25A42 is expressed in additional brain regions besides the hypothalamus and pons,^1^ leading to the assumption that SLC25A42 plays a central role in “basal brain function”.^1^ SLC25A42 is responsible for the uptake of coenzyme A (CoA) into the mitochondria in counter exchange with (deoxy)adenine nucleotides and adenosine 3′,5′‐diphosphate (PAP).^2^, ^3^

**FIGURE 1:**
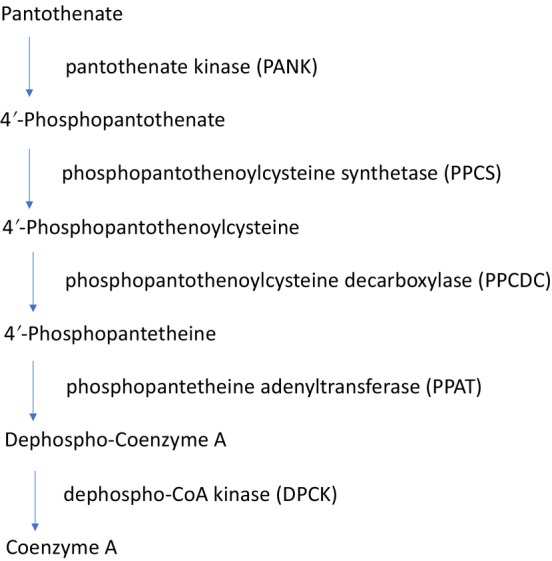
De novo biosynthesis of CoA. In the first enzymatic step, intracellular pantothenate is phosphorylated to 4′‐phosphopantothenate by pantothenate kinase (PANK). In the second step, 4′‐phosphopantothenate reacts with cysteine and ATP to form 4′‐phosphopantothenoylcysteine using phosphopantothenoylcysteine synthetase (PPCS). The third step includes decarboxylation of phosphopantothenoylcysteine by phosphopantothenoylcysteine decarboxylase (PPCDC), whereby 4′‐phosphopantetheine is formed. CoA synthase (COASY) carries out two enzymatic activities in the fourth and fifth steps. Initially, 4′‐phosphopantetheine is converted into dephospho‐CoA by 4′‐phosphopantetheine adenyltransferase (PPAT). Subsequently, dephospho‐CoA kinase (DPCK) produces CoA.^4^

CoA is a critical cofactor for more than 100 metabolic pathways, including the citrate cycle and the metabolism of fats, carbohydrates, and proteins.^4^ CoA is synthesized starting from pantothenate (vitamin B_5_), cysteine, and adenosinetriphosphate (ATP) in five well‐known enzymatic steps (Figure 1).^4^, ^5^, ^6^

Once formed, CoA can be transported across the inner mitochondrial membrane in exchange with 3′,5’‐ADP (PAP).^4^

To date, 21 cases with SLC25A42 deficiency (OMIM #618416) have been identified, mainly of Arab‐descend. Nineteen out of 21 cases carry the homozygous missense mutation c.871A > G, p.Asn291Asp^7^, ^8^, ^9^, ^10^; 1 case carries the homozygous splice change c.380 + 2 T > A, p(?),^9^ and a most recent case carries the homozygous c.523_526delATCC; p.Ile175Alafs*8 variant.^8^

Our study reports on the identification of new biallelic variants in *SLC25A42* in three siblings with varying clinical presentation, thus expanding the genetic and clinical spectrum of presentations of SLC25A42‐associated disorders. In addition, we provide evidence that SLC25A42 deficiency leads to reduced intracellular CoA levels in fibroblasts and that a high dose of pantothenic acid can make up for the deficit. Based on the in vitro results, high doses of pantothenic acid were supplied to the patients.

## 2 CASE REPORTS

Case 1, 2, and 3 are siblings born from unrelated healthy parents of German descent.

Case 1 died at 7 years of age. He was born at 41 weeks of gestation by cesarean section after an uneventful pregnancy. He showed normal development in the first 5 months of life. At 6 months of age, following a bronchopulmonary infection, the boy developed severe trunk muscle weakness, lost head control and developed dystonic‐ataxic movements of the extremities, tongue and pharyngeal muscles included. Abnormal lactate values (up to 5.9 mmol/L) and constant ketonuria were recorded. At 7 months of age, T2W and FLAIR brain magnetic resonance imaging (MRI) scans revealed symmetrical alterations in the putamen, on both sides. Over the years, multiple febrile infections led to metabolic decompensations with lactic acidosis. Case 1 became severely disabled and wheelchair‐bound. He could not speak although seemed to fully understand. At the age of 7 years, he caught an infection of unclear etiology, and his body temperature increased to 41.4°C within 90 min. On admission, Case 1 showed elevated leukocytes (34 700/μL. reference: 4.31–11.0/μL), normal CRP (<0.5 mg/L), and elevated IL‐6 (977 pg/mL, reference: <10 pg/mL). Volume substitution, antipyresis, and buffering with sodium bicarbonate were initiated. Antibiotic treatment with ampicillin‐sulbactam was given. Case 1 received a therapy consisting of carnitine, vitamins B and C, coenzyme Q, and a low dose of parenteral lipids. Case 1 showed severe metabolic lactic acidosis (lactate up to 13.5 mmol/L, pH 7.178/base excess −16.8) and within 24 h, the first signs of multiple organ failure with oliguria, disseminated intravascular coagulation (DIC), and rising transaminases (glutamate‐oxalacetate transaminase [GOT] up to max. 6525 U/L, reference: <50 U/L, Glutamate pyruvate transaminase [GPT] 1239 U/L, reference: <44.0 U/L) appeared. Lactate dehydrogenase (LDH) increased to 7029 U/L (reference: 129–222 U/L). Persistent coma, solitary respiratory pauses, and extensor spasms with loss of brainstem reflexes with light‐rigid and dilated pupils were observed. The patient died of secondary brain edema following the significant metabolic decompensation caused by the febrile infection.

Case 2 was healthy until the age of 11 years. Following a febrile infection, she developed swallowing problems and mild weakness, without remarkable findings in the pulmonological workup. Transient ketonuria was reported. Echocardiography, electrocardiogram (ECG), and electroencephalography (EEG) were normal. The patient kept having swallowing problems. At 16 years of age, the patient presented with repeated episodes of brief loss of consciousness. A brain MRI showed isolated, nonspecific FLAIR hyperintense lesions in the right inferior gyrus. The basal ganglia were unremarkable. Blood levels of lactate dehydrogenase (256 U/L, reference: 129–222 U/L) and lactate (2.6 mmol/L, reference: 0.4–2.0 mmol/L) were elevated.

Case 3 was born at 37 + 3 weeks of gestation as the mother developed preeclampsia during the last weeks of pregnancy. Besides that, the neonatal period was unremarkable.

At 3 months of age, the child developed high blood lactate (1.9 mmol/L, reference: 0.31–1.22 mmol/L) and slightly elevated creatine kinase (CK) (159 U/L, reference: up to 145 U/L). Repeated measurements confirmed the increase in lactate, whereby the blood count was unremarkable. Echocardiography revealed normal cardiac relations and dimensions and no cardiomyopathy. Head sonography showed inconspicuous basal ganglia. At 7 months of age, routine measurements of the lactate/creatinine ratio in the urine revealed an episodic increase (up to 55.6, reference: <0.2). After a mild febrile infection, she developed poor head control, difficulty in swallowing, shoulder girdle muscle weakness, and trunk instability. At 8 months of age, choreoathetosis and reduced muscle tone were displayed, with increased uncontrolled movement of the tongue and no reach for objects. During this period, acidosis, constant ketonuria, and blood lactate levels rose to 6.9 mmol/L. Venous blood gas analysis (BGA) showed: pH 7.255, BE −14.9 mmol/L, PC0_2_ 24.5 mmHg, and bicarbonate 12.4 mmol/L. There was no acute infection at that time, only a sore throat. Lower muscle tone, poor head control, and uncontrolled movements of the tongue and limbs persisted. During several febrile infections in childhood, Case 3 showed increased blood lactate. After each infectious episode blood lactate normalized. At 5 years of age, lactate levels decreased overall, and the girl showed progress in development functions. Case 3 presented developmental delay (sitting at 3 years, free running at 4–5 years, whole sentences at 8 years, and riding a bicycle at 12 years).

**FIGURE 2:**
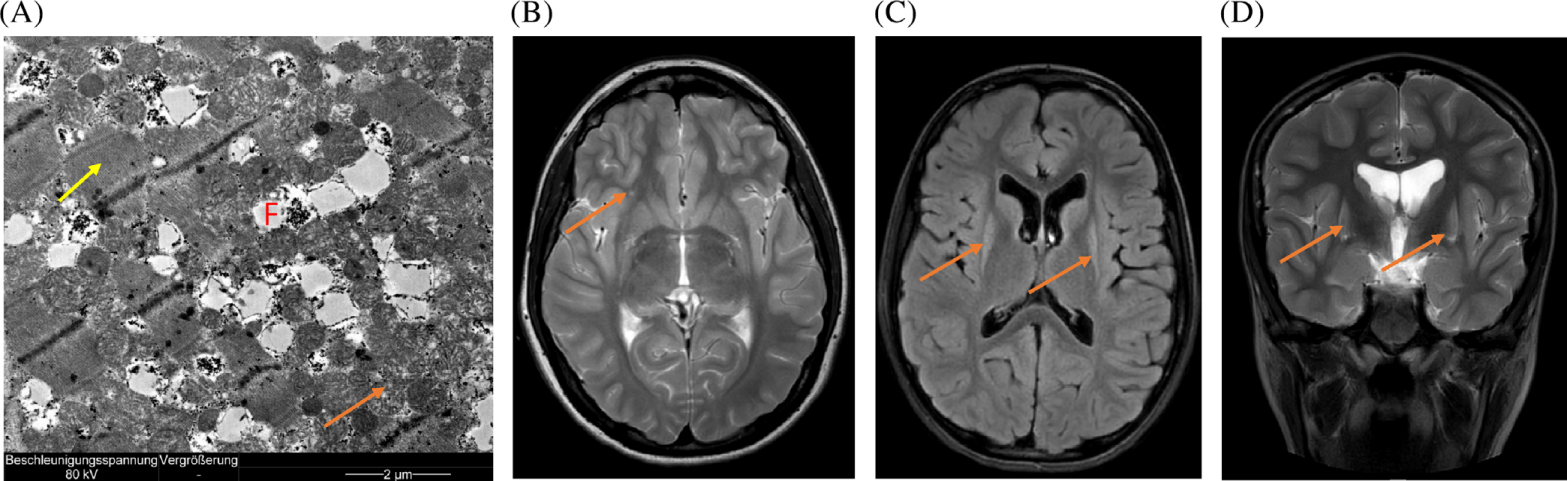
Electron microscopy of patients muscle biopsy and MR Image (Case 3 and 2). (A) Case 3: Sections of well‐structured muscle (yellow arrow) with a slight increase in the detection of mitochondria. (orange arrow) with a slight increase in fat storage (F). (B) Case 2: Axial T2 weighted MR image. The orange arrow depicts an unspecific hyperintense signal in the right inferior frontal gyrus. (C) Case 3: Axial FLAIR weighted MR image. The orange arrows depict the volume loss and hyperintense signal of the putamina. (D) Case 3: Coronal T2 weighted MR image. The orange arrows depict volume loss and hyperintense signal of the putamina.

At 1 year of age the brain MRI revealed normal basal ganglia; at 11 years, it showed bilateral, symmetrical T2 hyperintensity of the putamen with minor volume depression. The same changes were observed in the tail of caudate nucleus (Figure 2).

On the Wechsler Intelligence Scale for Children – Fifth Edition (WISC‐V, German translation, and adaptation), Case 3 scored a total IQ = 88 (90% confidence interval: 84–93) at 11 years and 11 months of age, corresponding to a percentile rank of 21.

Case 3 is now 13 years old. She shows a pronounced choreoathetosis, limited physical capacity, and cramps in the lower jaw muscles. A speech development disorder of expressive and receptive speech performance persists. Case 3 attends a mainstream school with the help of a full‐time integration worker.

## 3 MATERIALS AND METHODS

### 3.1 Genetic analysis

DNA was isolated from fibroblasts of Case 1 and whole‐exome sequencing (Illumina HiSeq2500 paired‐end [2 × 100 bp], Sure select v5 exom) was performed as previously described.^11^ Validation of the variants in *SLC25A42* and segregation analysis were performed on the genomic DNA of the three affected individuals and their parents by conventional Sanger sequencing (BigDye Terminator v3.1, Applied Biosystems) as outlined in.^12^

### 3.2 Reverse transcription‐polymerase chain reaction (RT‐PCR)

To detect possible nonsense‐mediated decay (NMD) of the *SLC25A42* transcript, reverse transcription (RT) of the mRNA and amplification by PCR of the region encompassing exons 5 to 8 was performed on the total RNA of Case 3 and her mother. RNA was isolated from EDTA blood with the PAXGene Blood RNA system (PreAnalytiX GmbH) followed by synthesis of cDNA (complementary DNA) using SuperScript™ IV reverse transcriptase (Invitrogen) as outlined in the manufacturer's protocol. Sequencing primers spanning exons 5–8 were designed with the open‐source Primer3 and BLAST software. Primers are available on request. PCR amplification was carried out as described in Würde et al.^13^ and amplicons were Sanger sequenced using BigDye Terminator v3.1 (Applied Biosystems).

### 3.3 Electron microscopy

Electron microscopy was performed on skeletal muscle tissue of Case 3. Tissue was fixed in 2.5% buffered glutaraldehyde, transferred to Sorensen's phosphate buffer (pH 7.2), dehydrated through ascending alcohols and embedded in epon. The epon blocks were trimmed, 1 μm‐thick sections were cut, stained with toluidine blue for epoxy semi‐thin sections, and examined by light microscopy to identify the regions of interest. Ultrathin sections were cut at the ultramicrotome with a diamond blade, mounted on a copper grid, stained with uranyl acetate (77 870, Serva) and Reynold's lead citrate and examined using transmission electron microscopy.^14^

### 3.4 Substrate oxidation rates and OXPHOS enzyme measurement

Measurements of substrate oxidation rates and activities of the respiratory chain enzymes were performed on fibroblasts of Case 1 as described earlier^15^ and fibroblasts of Case 3 as outlined in Ref. 16

### 3.5 Oxygen consumption rates (OCR)

OCR in fibroblasts of a healthy individual and Case 3 were determined using the Seahorse XFe^96^ Analyzer (Seahorse Bioscience). About 15 000 cells were seeded on XF Cell Culture Microplates (Seahorse Bioscience) in standard growth medium and incubated overnight at 37°C and 5% CO_2_. The next day, the medium was replaced with the Seahorse XF base medium (#103193–100; Agilent), supplemented with 10 mM glucose, 2 mM glutamine, and 1 mM pyruvate or 10 mM glucose, 2 mM glutamine and 0.1 mM palmitate‐BSA. Hence, the plate was incubated at 37°C without CO_2_ for 1 h. OCR was monitored upon serial injections of 2 μM oligomycin, 1 μM FCCP, and a 1 μM rotenone/antimycin A mixture. The concentration of 1 μM for FCCP (carbonyl cyanide‐4‐(trifluoromethoxy) phenylhydrazone) was determined in separate titration experiments to be optimal in uncoupling mitochondrial respiration (data not shown). OCR was normalized to the total protein amount by using a BCA assay.

### 3.6 Treatment of fibroblasts with pantothenic acid and CoA measurement

Fibroblasts were seeded on a 96 well‐plate at 800 cells/well density in 100 μL cell culture medium. 500 μM pantothenic acid (Sigma, P5155) was added to the cells 2 h after seeding. Five days after the treatment, cells were washed with PBS and lysed with 100 μL of mammalian lysis buffer (Abcam, ab179835). 50 μL of cellular lysate was used for the fluorometric evaluation of total cellular CoA (Abcam, ab138889), and values were normalized to the total protein amount measured using Bradford reagent (Bio‐Rad, #5000001).

## 4 RESULTS

### 4.1 Genetic analysis

**FIGURE 3:**
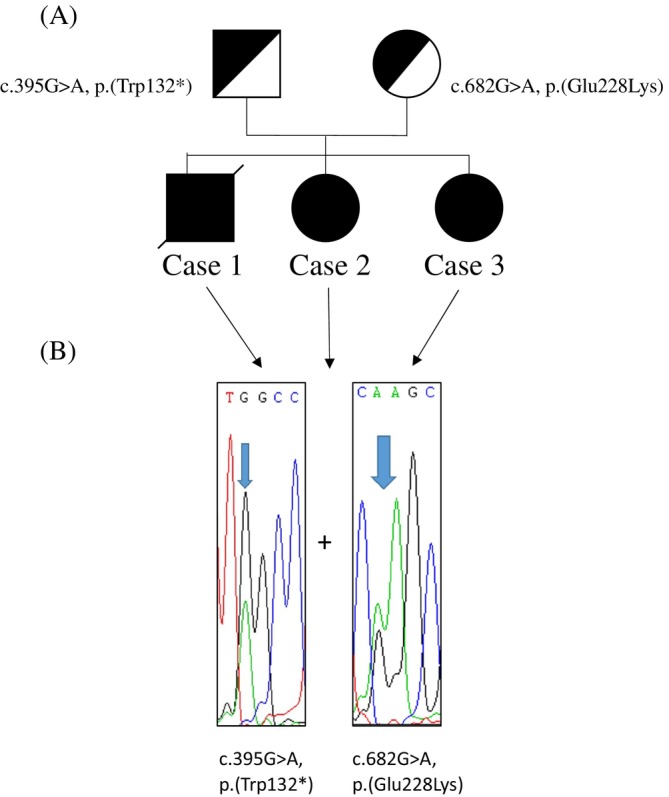
Investigated family. (A) Pedigree. (B) Electropherogram showing the sequencing results in Cases 1–3. All patients are compound heterozygous for c.395G > A, p.Trp132* and c.682G > A, p.Glu228Lys in *SLC25A42*.

Exome sequencing in Case 1 identified the compound heterozygous c.[395G > A];[682G > A], p.[Glu228Lys];[Trp132*] variants in *SLC25A42* (NM_178526.5). In GNOMAD, the variant c.682G > A could be identified four times in 124 370 alleles (allele frequency 0.003%, 4× heterozygous, 0× homozygous). Sanger sequencing confirmed the presence of both variants in the heterozygous state in the other two affected individuals, Cases 1 and 2. The mother was heterozygous only for the c.682G > A variant, while the father was heterozygous for the c.395G > A (Figure 3). Both variations are not described in the literature.

### 4.2 Reverse transcription‐polymerase chain reaction (RT‐PCR)

In Case 3, Sanger sequencing of the amplicons spanning exons 5 to 8, showed normal junction of exons 5–6, 6–7, and 7–8. Furthermore, it confirmed the presence of the variants 395G > A and 682G > A also at the cDNA level. In the mother only the c.682G > A variant was expressed in the heterozygous state (Figure 3).

### 4.3 Substrate oxidation rates and OXPHOS enzyme measurement

Substrate oxidation rates and OXPHOS enzyme measurement were performed both in fibroblasts and muscle homogenates of Cases 1 and 3. Case 1 was biopsied at the age of 2.5 years and 4.5 years, while Case 3 was biopsied at the age of 8 months and 3.5 years.

At the initial muscle biopsy at the age of 2.5 years, Case 1 presented with significantly reduced substrate oxidation rates in fresh muscle cells compared to control values, and an ATP production rate of only 15% compared to the lowest control value. A mild reduction of complex II and complex II + III was also noted. At a later biopsy at the age of 4.5 years, a fibroblast culture was established, and no defect in oxidative phosphorylation was evidenced (Table S1).

At the initial muscle biopsy at the age of 8 months, Case 3 presented with strongly reduced mitochondrial energy‐generating capacity in the muscle biopsy, combined with deficiencies of multiple respiratory chain enzymes. At the second biopsy, taken at 3.5 years of age, the reduced mitochondrial energy generating capacity was confirmed while the respiratory chain enzyme activities were normal (Table S2).

### 4.4 Seahorse results

**FIGURE 4:**
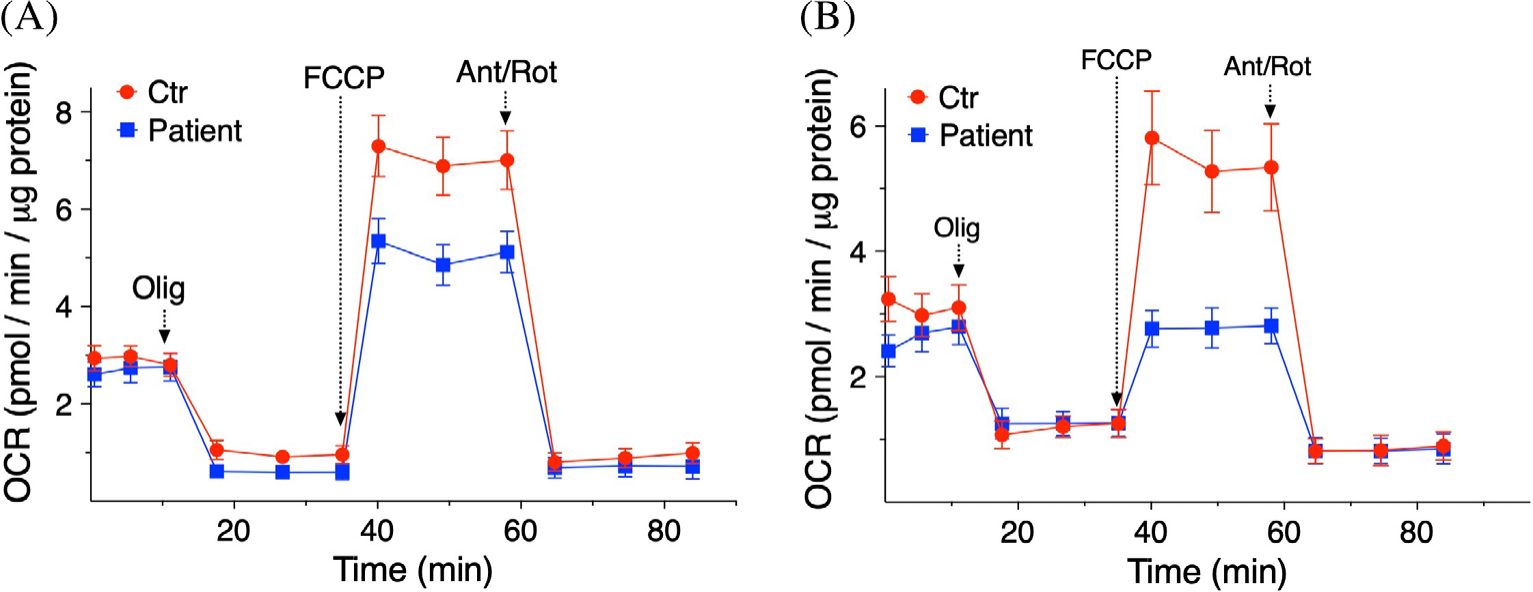
OCR in fibroblasts. OCR was measured with an XF96 extracellular flux analyzer (Seahorse). Cells were incubated for 1 h in base medium supplemented with glucose (10 mM), glutamine (2 mM) and pyruvate (1 mM) (A), or glucose (10 mM), glutamine (2 mM) and palmitate‐BSA (0.1 mM) (B). Cells were exposed to sequential additions of 2 μM oligomycin (Olig), 1 μM FCCP, and 1 μM antimycin A + 1 μM rotenone (Ant/Rot). OCR data are mean values ± SD from three independent experiments each including 5–6 replicates per cell type.

Control and fibroblasts of Case 3 had comparable basal respiration, whereas the maximal respiration was significantly lower in the patient (Figure 4A), above all when palmitate was used as a source of energy (Figure 4B). This suggests a mitochondrial respiration deficit in the patient, in particular, when the need for CoA availability increases (as in the presence of palmitate).

### 4.5 Electron microscopy

The skeletal muscle biopsy of Case 3 at 6 months and 3 years of age revealed a well‐structured muscle with numerous mitochondria and a slight increase in fat storage (Figure 2).

### 4.6 CoA measurement and in vitro treatment with pantothenic acid

CoA measurement in fibroblasts from patients Case 1 and Case 3 revealed a significant decrease in cellular CoA in fibroblasts compared to the level of CoA in commercial cells from a healthy individual (Lonza, NHDF, #CC‐2509). As a positive control for the measurement fibroblasts from a previously described patient with SLC25A42 deficiency were used.^9^

**FIGURE 5:**
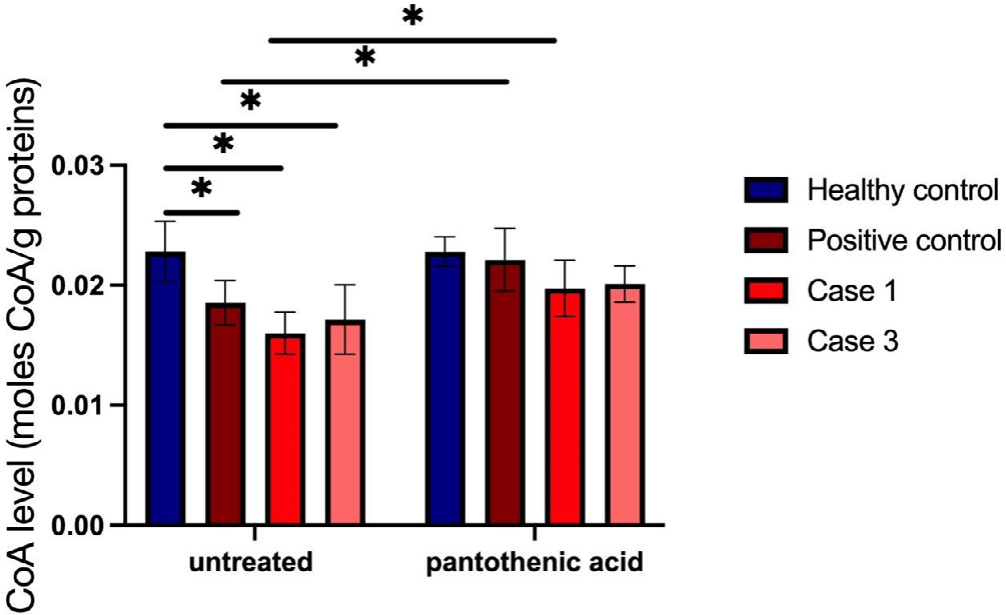
CoA measurement in fibroblasts. The endogenous level of CoA was measured in fibroblasts from a healthy individual, a patient with SLC25A42 mutation already reported to be pathogenic (Case 2, Iuso et al., 2019), and the two cases from the current study (Case 1, Case 3) before and after supplementation of cells with pantothenic acid. Results are mean ± SD of *n* = 2 independent experiments and *n* = 6 technical replicates. *p* values were calculated with an independent sample *t*‐test. *All *p* values were two‐sided with a significance level of 0.05.

After the in vitro treatment with pantothenic acid, the level of CoA increased significantly in fibroblasts from patient Case 3 and in the positive control. In fibroblasts from patient Case 1, there was a trend towards a CoA increase, although the increase did not reach the statistical significance threshold of *p* = 0.05 (Figure 5).

### 4.7 Treatment with pantothenic acid

At 13 years of age, Case 3 received oral administration of 250 mg of pantothenic acid twice a day (25 mg/kg/day) over a 6‐month period. The intake was well tolerated, and no side effects were observed. Pantothenic acid normalized the lactate values (from 2.4 to 1.03 mmol/L) and halted cramps in the lower jaw muscles which had followed an episodic increase of lactate to 2.09 mmol/L.

## 5 DISCUSSION

SLC25A42 deficiency is characterized by high clinical variability, ranging from almost asymptomatic cases to cases with severe metabolic decompensation and metabolic acidosis requiring intensive care.^8^

Our study confirms published evidence in terms of clinical variability and indicates that striking different phenotypes can also exist among siblings: Case 1 became wheelchair‐bound and steadily deteriorated after an infection‐triggered metabolic decompensation that culminated with the patient's death; Case 3 had a metabolic decompensation in early childhood and currently presents with choreoathetosis movements; Case 2 showed first signs of diseases at the age of 11 years and now lives an almost normal life.

The MRI findings in Cases 1 and 3 also fit the findings from published reports, as both cases showed abnormalities in the putamen,^7^, ^8^, ^9^ while MRI findings in Case 2 presented one nonspecific FLAIR hyperintense lesion in the right inferior gyrus, probably unspecific, and not yet associated with SLC25A42 deficiency.

From the functional point of view, our data suggest that SLC25A42 is essential when patients are in high demand of energy, for instance, to respond to infections with febrile progression, as in our cases. Despite only being speculative, it is likely that neurological impairment arises upon a continuous unfulfilled energy demand.

In particular, the utilization of fatty acids as source of energy seems to be affected by SLC25A42 mutations. Patients' fibroblasts displayed reduced levels of CoA. As CoA is needed for the esterification of fatty acids used by mitochondria for the ß‐oxidation,^17^ reduced levels of CoA can indirectly affect b‐oxidation, as shown by the impaired utilization of palmitate in the functional mitochondrial assays. This preliminary finding, derived from extracellular flux analyses on fibroblasts from a single case, suggests that a low‐fat diet might aid in maintaining energy homeostasis in individuals with SLC25A42 deficiency. However, due to the limited sample size, further research is necessary to confirm this observation.

Since there are no targeted therapies for SLC25A42 deficiency, we decided to supply pantothenic acid, the water‐soluble vitamin converted to CoA through a conserved biosynthetic process, to the patients in the attempt to rescue CoA levels.

The amount of pantothenic acid provided to the patient was 100 times higher than the normal daily requirement (5 mg/d in 14‐year‐old individuals^10^). While we recorded the stabilization of the clinical conditions (no worsening was reported after pantothenic acid supplementation), we could not observe any amelioration/reversing of the phenotype. It is unclear if this lack of substantial improvement was due to insufficient bioavailability of pantothenic acid (biochemical markers to quantitatively track pantothenic acid intake are lacking) or instability of pantothenic acid, degraded in serum into pantothenate and cysteamine by vanins.^18^, ^19^

Approaches that could be exploited in the future to increase the levels of CoA are (i) the use of 4′‐phosphopantetheine in place of pantothenic acid; 4′‐phosphopantetheine is a stable intermediate of the CoA synthesis, passing the membranes via passive diffusion.^19^ Unfortunately, it is not yet available on the market, and (ii) the modulation of the microbiome.^2^ Sibon et al. showed that in Drosophila the gut microbiome contributes to modulating the levels of CoA in the host.^20^ If this mechanism would also happen in men, it could explain the variable phenotypes associated with SLC25A42 deficiency, also among siblings carrying the same pathogenic variants and sharing a similar genetic background.

While this study provides valuable insights into SLC25A42 deficiency and a potential treatment approach, there are some limitations. The clinical experience with the treatment is limited to one patient, and the relationship between the dose used in the patient and the dose used in the in vitro experiment is not aligned. Additionally, there is a lack of distinction between mitochondrial and cytosolic CoA pools, so it cannot be stated whether the cytosolic or mitochondrial CoA pool increased. Further investigations are needed to determine if an increase in cytosolic CoA leads to an increase in mitochondrial CoA. In conclusion, further research is needed to address these limitations and expand our understanding of this condition and its management.

## AUTHOR CONTRIBUTIONS

K. Heckmann: collecting clinical data and samples, acquisition and analysis of data, drafting and revision of the manuscript. M. Grüneberg, A. Seelhöfer, S. Rust, J. Reunert: acquisition and analysis of data, Genetic analysis, revision of the manuscript. T. Marquardt: supervision, data acquisition and interpretation, revision of the manuscript. A. Iuso: in vitro treatment with pantothenic acid, CoA measurement, and revision of the manuscript. G. Fiermonte: Seahorse experiment, revision of the manuscript. E. Paradies: molecular analysis of Seahorse experiment, revision of the manuscript. C. Piazzolla: Seahorse experiment, revision of the manuscript. M. Manoj: Analysis and description of MRI Images, revision of the manuscript.

## FUNDING INFORMATION

Giuseppe Fiermonte, Carmela Piazolla and Eleonora Paradies were supported by the Italian Ministero dell'Istruzione, dell'Università e della Ricerca, MIUR, 2017PAB8EM.

## CONFLICT OF INTEREST STATEMENT

Katharina Heckmann, Arcangela Iuso, Marianne Grueneberg, Anja Seelhoefer, Stefan Rust, Janine Reunert, Giuseppe Fiermonte, Eleonora Paradies, Carmen Piazolla, Mannil Manoj and Thorsten Marquardt declare that they have no conflict of interest.

## ETHICS STATEMENT

All the followed procedures were in accordance with the ethical standards of the responsible committee on human experimentation (institutional and national) and with the Helsinki Declaration of 1975, as revised in 2000 (5).

## PATIENT'S CONSENT

Informed consent was obtained from all patients and their parents included in the study. The proof is available upon request. Approval for investigations was obtained from the local Bioethics Committee (Number: 2019‐199‐f‐s Approval date: 07. February 2020).

## ANIMAL RIGHTS

This article does not contain data generated using animal models.

## Supporting information


**Table S1.** Analysis of substrate oxidation rates and respiratory chain enzymes: Case 1.

**Table S2.** Analysis of substrate oxidation rates and respiratory chain enzymes: Case 3.

## Data Availability

All data presented in this article are available from the corresponding author upon reasonable request.
