# Correction to “Expanding the Genetic and Clinical Spectrum of SLC25A42 Associated Disorders and Testing of Pantothenic Acid to Improve CoA Level In Vitro”

**DOI:** 10.1002/jmd2.70002

**Published:** 2025-02-10

K. Heckmann , A. Iuso , J. Reunert et al., “Expanding the Genetic and Clinical Spectrum of SLC25A42‐Associated Disorders and Testing of Pantothenic Acid to Improve CoA Level In Vitro,” JIMD Reports 65 (2024): 417–425, 10.1002/jmd2.12441.39512436 PMC11540568

Affiliation of Eleonora Paradies was listed incorrectly. Correct affiliation is:

Katharina Heckmann^1^, Arcangela Iuso^2,3^, Janine Reunert^1^, Marianne Grüneberg^1^, Anja Seelhöfer^1^, Stephan Rust^1^, Giuseppe Fiermonte^4^, Eleonora Paradies^5^, Carmela Piazzolla^4^, Manoj Mannil^6^, Thorsten Marquardt^1^.

^1^ Department of General Pediatrics, University Hospital, Münster, Germany.

^2^ Institute of Neurogenomics, Helmholtz Zentrum München, Neuherberg, Germany.

^3^ Institute of Human Genetics, Technical University of Munich, Germany.

^4^ Department of Biosciences, Biotechnologies and Biopharmaceutics, University of Bari, Italy.

^5^ National Research Council, Institute of Biomembranes, Bioenergetics and Molecular Biotechnology (IBIOM), Bari, Italy.

^6^ Clinic of Radiology, University Hospital Münster, Münster, Germany.

We apologize for this error.

